# Implementation and Assessment of an Intervention to Debias Adolescents against Causal Illusions

**DOI:** 10.1371/journal.pone.0071303

**Published:** 2013-08-14

**Authors:** Itxaso Barberia, Fernando Blanco, Carmelo P. Cubillas, Helena Matute

**Affiliations:** Departamento de Fundamentos y Métodos de la Psicología, Universidad de Deusto, Bilbao, Spain; Universidad de Granada, Spain

## Abstract

Researchers have warned that causal illusions are at the root of many superstitious beliefs and fuel many people’s faith in pseudoscience, thus generating significant suffering in modern society. Therefore, it is critical that we understand the mechanisms by which these illusions develop and persist. A vast amount of research in psychology has investigated these mechanisms, but little work has been done on the extent to which it is possible to debias individuals against causal illusions. We present an intervention in which a sample of adolescents was introduced to the concept of experimental control, focusing on the need to consider the base rate of the outcome variable in order to determine if a causal relationship exists. The effectiveness of the intervention was measured using a standard contingency learning task that involved fake medicines that typically produce causal illusions. Half of the participants performed the contingency learning task before participating in the educational intervention (the control group), and the other half performed the task after they had completed the intervention (the experimental group). The participants in the experimental group made more realistic causal judgments than did those in the control group, which served as a baseline. To the best of our knowledge, this is the first evidence-based educational intervention that could be easily implemented to reduce causal illusions and the many problems associated with them, such as superstitions and belief in pseudoscience.

## Introduction

Despite the exponential development of scientific research in recent decades, the sad truth is that many people still hold a vast number of unrealistic and irrational beliefs about the functioning of the world. Some of these beliefs are clearly eccentric and openly violate our present knowledge about the laws of nature, including superstitions related to supernatural forces such as omens, witchcraft, astrology, and psychic powers. Moore [1] reported that belief in psychics and paranormal activity is worryingly prevalent in the American population. For example, 32% of the people interviewed in 2005 believed in ghosts, 37% thought that houses can be haunted, and 21% believed that witches exist (see also [2]).

Misbeliefs also underlie many pseudoscientific practices, which are especially dangerous because they “possess the superficial appearance of science but lack its substance” ([3] p. 1216). These practices are intentionally presented as scientific, even if they do not meet the minimum acceptable standards for science. This is the case for many so-called “alternative medicines”, such as homeopathy. According to the Special Eurobarometer on Science and Technology [4], 34% of Europeans consider homeopathy to be “scientific”. Meanwhile, the purported therapeutic mechanism of homeopathic products is implausible, and research shows that their alleged healing effects may be attributable solely to the placebo effect [5]. Nevertheless, many people use these products, sometimes substituting them for treatments with demonstrated efficacy [6]. It should be obvious that superstitious and pseudoscientific beliefs become extremely worrisome when they begin to drive people’s decisions about many important areas of their daily lives. The effects range from the expenses paid to fortune-tellers or clairvoyants to health risks of ineffective treatments for a variety of illnesses.

Interestingly, one phenomenon that is central to these unrealistic beliefs is the fact that people sometimes develop illusions of causality, that is, they perceive the existence of causal links between events that are actually uncorrelated [7], [8]. Our cognitive system has evolved to sensitively detect causal relationships in the environment, as this ability is fundamental to predict future events and adjust our behavior accordingly. However, under certain conditions, the very same cognitive architecture that encourages us to search for causal patterns may lead us to erroneously perceive causal links that do not actually exist. These false perceptions of causality may be the mechanism underlying the emergence and maintenance of many types of irrational beliefs, such as superstitions and belief in pseudoscience. These illusions could also be the basis of many types of group stereotypes [9] and may promote ideological extremism [10] hence contributing to intergroup conflict and suffering throughout the world.

A fundamental difficulty of causal induction, as acknowledged by philosophers such as Hume [11], is that causality is not directly observable and must therefore be inferred from empirical evidence. One of the primary empirical inputs for causality is the correlation or contingency between the potential cause and the outcome of interest. Given one binary cause and one binary outcome, the contingency can be easily formalized by using the Δp index [12], [13], which is the difference between the probability of the outcome given the presence of the potential cause and the probability of the outcome given its absence: Δp = P(Outcome|Cause) - P(Outcome|∼Cause). The result of this subtraction determines the contribution of the potential cause to the occurrence of the outcome, providing information about the generative, preventive, or null influence of the former on the latter. Although more sophisticated rules than Δp have been formulated as a normative standard for assessing causal strength [14], they usually include the same contrast between the two mentioned conditional probabilities. Furthermore, most normative indexes yield the same result when assessing null contingencies, that is, situations in which P(Outcome|Cause)  =  P(Outcome|∼Cause), which are the major focus of this paper.

Much as it could happen in real world, the participants in a typical causal learning experiment are initially presented with two events that might be causally related (the target cause and the outcome). Imagine that the hypothesized relationship involves a certain herb as a potentially effective remedy for headaches. Next, the participants are sequentially exposed to several observations (in this example, several fictitious patients) in which the presence and the absence of the potential cause and the outcome are combined to generate four different possible situations: (a) the herb (i.e., the potential cause) is ingested, and relief (i.e., the outcome) occurs; (b) the herb is taken, and relief is not observed; (c) the herb is not ingested, but relief occurs; and (d) the herb is not taken, and relief is not observed. The contingency between the potential cause and the outcome is experimentally manipulated by determining the frequency of each of these four types of event. After being exposed to a number of these fictitious cases during the training phase, the participants are asked to evaluate the extent to which the herb is actually able to produce relief (i.e., to judge the strength of the causal relationship between the potential cause and the outcome under study). Although some studies have demonstrated that participants’ causal perception is in fact sensitive to the actual contingency [15], [16], [17], systematic biases have been detected as well.

One extensively studied bias is the *illusion of causality* or *causal illusion* cited above (note that we will use the general term *illusion of causality* or *causal illusion* to refer to what some authors have called the *illusion of control*. We assume that the latter is simply a specific case of the former). This bias refers to the perception of a causal relationship when the target event and the outcome occur together by mere chance, but are actually independent of each other. That is, the probability of the outcome is the same regardless of the presence or absence of the potential cause, P(Outcome|Cause) = P(Outcome|∼Cause), and, therefore the contingency is zero. One fundamental factor that modulates the magnitude of this illusion is the probability of the occurrence of the outcome, P(Outcome). When the outcome occurs frequently, the illusory perception of a causal link is facilitated [18], [19], [20], [21], [22], [23].

Relevant to the present work, the probability of the potential cause, P(Cause) also influences the illusion of causality. More specifically, as P(Cause) increases, so it does the misperception of the effectiveness of the potential cause, especially when the outcome also occurs relatively frequently [20], [24], [25]. Significantly, in those experiments, the contingency is always set to zero; therefore, the probability of relief will be similar regardless of whether the herb is administered or not. However, if the P(Outcome) is high, many patients will recover from their headaches independent of the administration of the herb. In these circumstances, any potential treatment (such as the herb in our example) will have a strong chance of co-occurring (or occurring close in time) with healing. Moreover, the number of co-occurrences will increase as P(Cause) increases. These coincidences may be largely responsible for the increased perception of a causal relationship. In fact, as noted by many researchers [26], [27], [28], [29], [30], [31], [32], causal impressions may be guided by heuristics that give more weight to cause-outcome coincidences (i.e., instances that seem to confirm the relationship) than to outcome occurrences that could not have been generated by the potential cause (but see [33] for an explanation of how, under certain assumptions, it could be normative to weight coincidences more heavily than the rest of event types).

On this basis, one way to diminish causal illusions may be to decrease the number of accidental cause-outcome coincidences to which people are exposed. This goal might be reached by decreasing either the P(Outcome) or the P(Cause). Unfortunately, the P(Outcome) is usually out of individual and institutional control in real life (e.g., it is not possible to change the prevalence of conditions such as headaches). However, the P(Cause) is frequently subject to individual decisions and can therefore be modified. In this sense, previous research on causal illusions has sometimes employed contingency learning tasks in which the participants are allowed to decide, for each observation, whether they want to introduce the target cause or not, and subsequently observe if the outcome occurs [18], [24], [25]. In these active tasks in which the participant’s behavior directly determines the proportion of trials in which the cause is present, instructional manipulations can reduce the illusion of causality by encouraging the participant to introduce the target cause in approximately half of the trials [20], [25], [34]. However, when the instructions simply request that the participants try to obtain the outcome as often as possible [24], [25], the participants’ natural tendency seems to be to introduce the potential cause a relatively high number of times. For example, Blanco et al. [24] found that the participants in their study introduced the potential cause in more than half of the trials and that this behavior became more marked as the experiment progressed. Although Blanco et al. [24] did not report this specific statistical analysis, we analyzed their data and observed that their participants administered the potential cause more often than that expected by chance. A comparison against a theoretical P(Cause) of 0.5 showed that the difference was statistically significant, *t*(81) = 3.86, *p*<.001, for Experiment 1 and *t*(91) = 7.63, *p*<.001, for Experiment 2.

This tendency to focus on the instances of the reality in which the potential cause is present might be related to a more general bias in people’s information sampling strategies. To this respect, several authors have called attention to an effect that is sometimes called the *positivity bias* or *positive testing strategy* [29], [35], [36]. This effect refers to the finding that, when testing the validity of a hypothesis, people predominately sample cases that would produce a positive outcome if the hypothesis were correct [37], [38], [39], [40], [41] (note that even though some of these findings were initially interpreted as indicating a *confirmation bias*, Klayman and Ha [38] subsequently argued that the preference for positive tests does not necessarily represent a bias towards confirmation, see also [37], [42]). For example, when testing the hypothesis that a person is an extrovert, people tend to ask more questions that refer to extraversion than questions that refer to introversion [40]. In other words, people preferentially ask questions that would receive an affirmative answer if the person were indeed an extravert. We propose that a similar hypothesis-testing strategy might operate in a typical causal learning experiment. If we are presented with the goal of determining whether an herb is an effective remedy for eliminating headaches, a positive testing strategy might involve predominately choosing to observe cases in which the herb is taken rather than cases in which the herb is not taken. A high P(Cause) value is the logical consequence of a positive testing strategy. If relief from headaches occurs at a high rate [i.e., if P(Outcome) is high] and we frequently ingest the herb when we experience the early symptoms of a headache [i.e., if P(Cause) is high], then recovery will frequently follow herb ingestion. Because we persevere in this behavior, we tend not to be exposed (or at least we are less often exposed) to information related to the actual rate of spontaneous remission. As a result, the impression that the herb is effective will persist.

A straightforward method of overcoming this natural tendency might involve encouraging people to recognize the importance of searching and considering information about the state of facts when the potential cause is absent. This is in some sense analogous to teaching people about the logic of experimentation in science, in which experimental and control groups are meticulously designed to assess the influence of the factor that is being tested, independent of any other confounding variables. Although this idea underlies many scientific educational programs [43], [44], [45], we know of no program that has tested the efficacy of this training in reducing subsequent causal illusions. Based on this idea, the goal of the present study was to develop and test an educational intervention that would reduce the tendency to illusory perceptions of causality by encouraging people to understand the importance of exposing themselves to more cause-absent control observations. The targets of the intervention were secondary school students. The decision of addressing the intervention to this population was both practical (i.e., students at that level are still immersed in educational contexts in which it is easier to intervene) and theoretical, as adult resistance to scientific thinking may arise soon in life [46].

### Overview of the Intervention

The intervention introduced the participants to the concept of contingency described above. In the intervention, the participants learned that comparing the probability of an outcome in the presence and the absence of the potential cause is the normative manner of assessing the empirical evidence for a hypothesized causal link. In this sense, the emphasis was placed on the idea that the rate of cause-outcome co-occurrence, or P(Outcome|Cause), is necessary to infer a generative causal relationship between a potential cause and an outcome but is certainly not sufficient. The base rate of the outcome, or P(Outcome|∼Cause), is also important to consider if one is to reach an appropriate conclusion. Understanding the necessity of considering this latter piece of information should encourage people to expose themselves to more instances in which the cause is absent. That is, to expose themselves to a lower P(Cause), which should, in turn, diminish the tendency to develop causal illusions.

We know of no other educational intervention that was designed to debias people against causal illusions. Indeed, relatively little work has been done to investigate the extent to which debiasing against a variety of cognitive biases is possible, and the results have been mixed (see [10], [47], [48] for reviews). Lilienfeld et al. [10] eloquently noted several potential barriers to successful debiasing interventions that should not be ignored. First, they suggested that people might not debias because they tend not to accept that their perspective is biased; this effect is called the “bias blind spot” or the “not me” bias [49], [50], [51]. Second, they alerted that interventions are more effective when people perceive that the bias to be corrected is relevant to their daily lives [47], [52]. Following Lilienfeld et al.'s [10] recommendations, we expected that we might improve the effectiveness of our debiasing intervention by first demonstrating to the participants how easily they might arrive at biased conclusions and how relevant these conclusions might be to their daily lives. To achieve this goal, the intervention included an initial section in which we tried to induce a causal illusion in our participants before proceeding to explain how to approach causal induction normatively.

The effectiveness of the intervention was measured by exposing the participants to a typical contingency learning task, as described in the Introduction. The participants were asked to determine the strength of a cause-outcome relationship through successive observations in which they could decide if they wanted to introduce the potential cause and then subsequently observed whether the outcome occurred. Half of the participants (the control group) performed the contingency learning task before the intervention was conducted, whereas the other half (the experimental group) performed the same task after the intervention was completed. Our hypothesis was that the intervention would improve the ability of the experimental participants to gather and evaluate empirical evidence for a potential causal relationship. More specifically, we expected that the participants who had been exposed to the intervention would show a decreased tendency to choose to observe cause-present observations [i.e., they would generate lower P(Cause) values] and would exhibit weaker causality illusions than the participants who performed the task before the intervention.

The computer task involved sequentially investigating two potential causal relationships. That is, the participants were asked to evaluate the effectiveness of a medicine not only in a zero contingency situation in which P(Outcome|Cause) = P(Outcome|∼Cause), but also in a second situation in which there was a generative relationship between the potential cause and the outcome, P(Outcome|Cause) >P(Outcome|∼Cause). The positive contingency condition served to control for the possibility that the intervention made the participants generally more skeptical about any potential cause-effect relationship, rather than improving their specific understanding of how causal effectiveness should be assessed and interpreted.

## Methods

### Ethics Statement

The ethical review committee of the University of Deusto approved this study. The intervention was offered to the Faculty of Engineering of the University of Deusto as a workshop on critical thinking that could be implemented as part of their larger set of activities in a summer camp on technology and robotics. The Faculty of Engineering was provided with detailed written information on the purpose, methods, and data treatment of the study before they invited us to participate in their programs. Only those minors whose parents, next of kin, guardians, or caretakers specifically requested in their general application to the summer camp to participate in our workshop (including its evaluation and statistical treatment and potential publication of the data) participated in the workshop and the present evaluation of its efficacy. The ethical review committee of the University of Deusto considered that the experimenters did not need to obtain an additional direct consent of the participants’ parents, but only that of the summer camp organizers, which was obtained verbally in agreement with the ethical review committee advice.

### Participants

Sixty-two secondary school students participated in the study. The control group and the experimental group both included 31 participants. Data were not recorded for two participants in the control group; therefore, they are not included in the analyses. Thus, the final N was 60. In addition, two participants in the control group failed to provide their ages. Among the participants who reported their ages, the average ages were 14.26 (*SEM = *0.30) and 14.84 (*SEM = *0.29) in the control and experimental groups, respectively. There were no significant age differences between the groups: *t*(56) = 1.38, *p = *.17.

### Procedure

The same intervention was repeated in four separate sessions, each involving different participants (a minimum of 12 participants and a maximum of 20 per session). The participants were randomly assigned to the control and experimental groups at the beginning of each session, and each group was placed in a different classroom. The only variation between the groups was the time at which the participants performed the contingency learning task: the control group performed the task before the intervention, and the experimental group performed the task after the intervention. The instructions for the contingency learning task were provided by the same experimenter for both groups.

#### Intervention

The intervention was divided into two phases. The first phase involved staging. The second phase involved an explanation of the appropriate manner of approaching everyday causal inference. Both phases were completed in a single session of approximately 80 minutes.

#### Phase 1

The goal of this phase was to generate a situation in which participants might be inclined to form a biased impression of the effectiveness of a target product. The product was a small rectangular piece of regular ferrite. However, the participants were told that the product was made of a brand new material that had recently been developed by a group of researchers. They were told that upon contact with human skin, the product stimulates the nervous system, improving both the physical and the intellectual abilities of its carrier. Mimicking the strategy used in pseudoscience [3], [49], we offered an explanation that was intentionally hyper-technical (i.e., we employed scientific jargon, using words such as *electromagnetism*, *cell*, *atom*, *nervous system*, and *activation*).

Once the alleged properties of the ferrite bar had been explained, the experimenter attached the product to the wrist of each of the participants. The participants were then asked to perform a series of tasks to experience the benefits of the product. First, they had to complete several paper-and-pencil tasks (e.g., solving mazes or crossing out all the consonants from a matrix of letters and numbers) as quickly as possible. Significantly, the participants always performed these tasks while wearing the ferrite bar. Therefore, they lacked a decisive control condition to which compare their performance. However, we tried to influence the participants’ perceptions regarding the effectiveness of the product by telling them after each exercise that the people who wore the product in previous tests reported that they had felt that they had performed the tasks especially well (e.g., when solving the mazes, they could determine the solution very quickly, as if their minds were faster than their hands).

In a second series of activities, the participants were presented with several physical exercises involving strength, stability and flexibility. The exercises were similar to those advertised on the webpages of popular performance-enhancing bracelets that have been proven to be bogus, such as Power Balance® (www.powerbalance.com/test-video, as cited by Porcari et al. [53]; this video has now been removed). Using a procedure similar to the procedure presented in these videos, we encouraged the participants to perform each exercise first without wearing the ferrite bar and immediately afterwards holding the product in one hand. Therefore, in this second series of activities, we did provide the participants with a control condition (i.e., they could compare their performance with and without the product). However, the control condition was intentionally suboptimal because the influence of the ferrite bar was confounded with the potential influence of warming up and learning from practice (i.e., the test with the product was always performed second). In fact, previous research has shown that the alleged effects of holographic bracelets disappear when the effect of order is controlled for [53], [54].

#### Phase 2

After the first staging phase and before we informed the participants that the product was fake, we introduced the participants to the concept of contingency as the correct way to infer causality from empirical information. Before introducing the idea of contingency, we suggested some examples in which the target cause was frequently followed by the outcome (see [55] for the additive benefit of employing both formal rules and examples in statistical reasoning training). For instance, we presented a situation similar to the herb-relief example described in the introduction and asked questions such as “If 80% of people who take the herb feel better, does this represent proof of the herb’s effectiveness?” These examples were used to emphasize the idea that even a high rate of cause-outcome coincidences does not guarantee the existence of a causal link. Moreover, we focused on the fact that if people experienced relief soon after taking the herb, they might feel inclined to continue using the herb in the future, thus depriving themselves of the possibility of observing whether relief was likely to occur spontaneously (i.e., without their taking the herb).

The participants were also briefly introduced to the importance of choosing a good control condition when testing causal links. We suggested that it is fundamental to compare the probability of the outcome in the presence and the absence of the potential cause while independently controlling for all of the other factors that could also affect the outcome. For example, the participants were invited to consider the case of a plant fertilizer that is tested on a farm in a rainy location. If the growth observed at this farm is compared with that observed at a farm that did not receive the fertilizer but is located in a drier place, we will not be able to determine if the superior growth of the plants that received the fertilizer is due to the fertilizer or to the differences in the climate. This example is analogous to the unclear boundary between the influence of the product and the influence of the practice in the physical exercises performed in the first phase of the intervention.

At the end of this phase, the participants were asked to judge, given the information they received, whether the method of testing the ferrite bar that was used in the first phase was adequate. After a discussion about the problems related to the cognitive tasks (i.e., a discussion of the lack of a control condition) and the physical activities (i.e., a discussion of the inadequacy of the control condition), we revealed the truth to the participants about the ineffectiveness of the ferrite bar.

#### Measurement (contingency learning task)

Participants in neither group were given any information about the purpose of the contingency learning task. In the case of the control group, they were told that, before starting with the workshop, they would be playing a computer game. Similarly, participants in the experimental group were told that the workshop was finished and that they would then play a computer game.

The procedure was similar to the conventional contingency learning paradigm used in the literature (e.g., [24]). It consisted of two different stages. In the first stage, the participants were asked to imagine that they were medical doctors. They were told that a fictitious medicine, Batatrim, could potentially provide relief for patients suffering from a fictitious illness called the Lindsay syndrome. They were asked to determine the extent to which the medicine was effective. To do so, the participants sequentially observed the records of 40 fictitious patients suffering from the syndrome and decided whether they wanted to administer the medicine to each patient. In each trial, after making the decision, they observed whether the patient was cured. After all 40 patients had been observed, the participants were asked to evaluate the effectiveness of the medicine on a scale ranging from 0 (ineffective) to 100 (entirely effective). In the second stage, the participants were presented with a second medicine, Dugetil, as a potential treatment for a second syndrome, Hamkaoman. The procedure was exactly the same as the procedure for the first medicine.

One of the medicines was presented in a zero contingency condition, P(Outcome|Cause) = P(Outcome|∼Cause), whereas the other was presented in a positive contingency condition, P(Outcome|Cause) >P(Outcome|∼Cause). In the zero contingency condition, 6 out of every 8 patients felt relief independent of the participants’ decision to administer the medicine (i.e., the probability of relief was.75 with or without the medicine). In the positive contingency condition, only 1 out of every 8 patients who did not receive the medicine felt relief, whereas 6 out of every 8 felt relief after taking the medicine (i.e., the probability of relief was.75 with the medicine and.125 without it). Therefore, the objective contingency was.625 in the positive contingency condition. Half of the participants from each group were exposed first to the zero contingency condition and then to the positive contingency condition. This order was reversed for the other half of the participants.

It is plausible to assume that the participants in the experimental group, who had just been deceived by the experimenters, might have been especially likely to feel suspicious when performing this task and might therefore have been less prone to assume the existence of a causal relationship, regardless of the evidence they encountered. The positive contingency condition was introduced to control for this potential confounding effect. We expected the participants in the experimental group to be more realistic in the zero contingency condition while retaining the ability to reach an accurate conclusion about the positive contingency condition.

## Results

**Figure 1:**
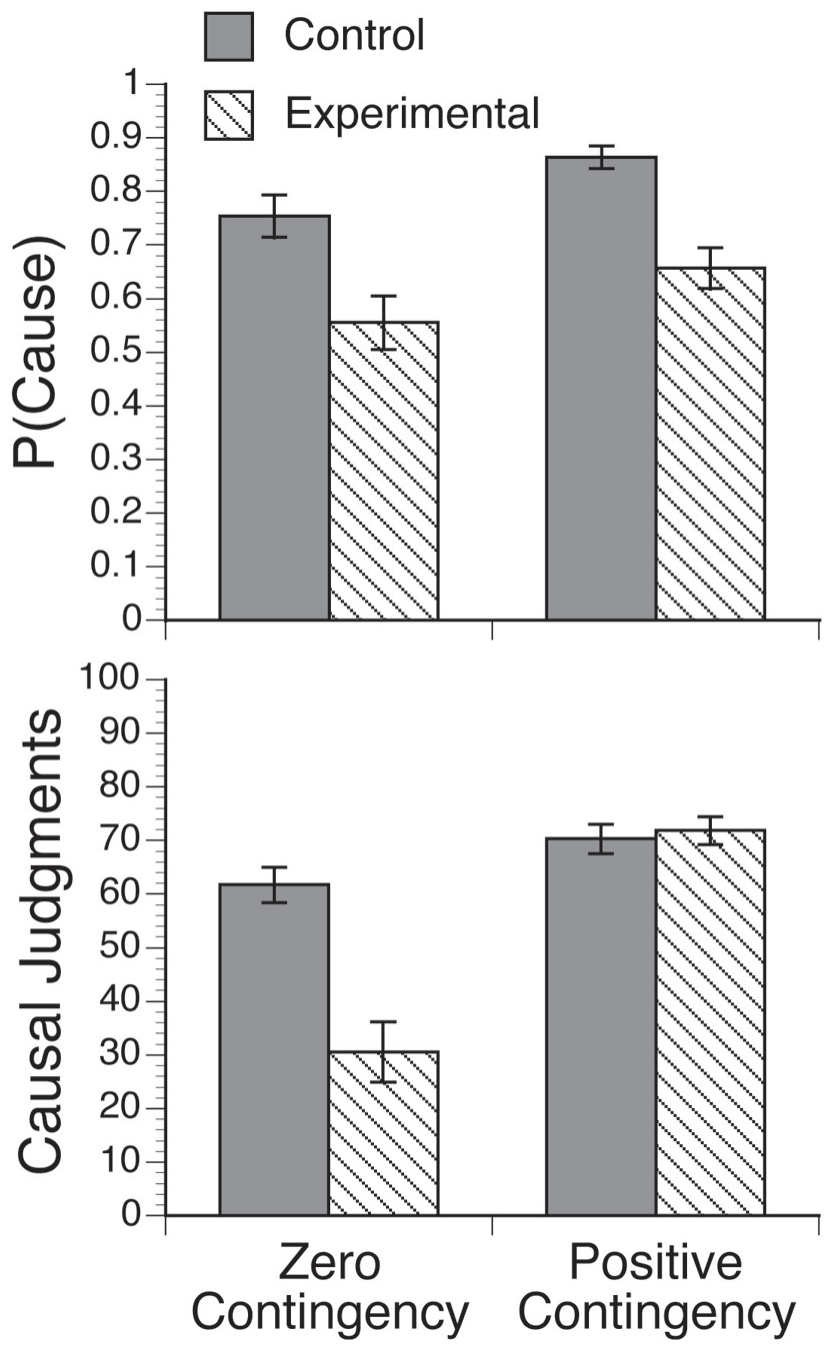
Mean P(Cause) (top panel) and mean causal judgments (bottom panel). The top panel represents the mean proportion of trials in which the participants from the control and experimental groups decided to administer the medicine in the zero contingency and positive contingency conditions. The bottom panel represents the mean judgments regarding the effectiveness of the medicines in the zero contingency and positive contingency conditions. The filled bars refer to the participants in the control group, and the striped bars refer to the participants in the experimental group. The error bars represent the standard error of the mean.

The top panel of Figure 1 shows the proportion of trials in which the participants from each group chose to administer the medicine [i.e., this figure represents the P(Cause) values]. The mean P(Cause) in the zero contingency condition was.75 (*SEM = *.04) for the control group and.56 (*SEM = *.05) for the experimental group, whereas the mean P(Cause) in the positive contingency condition was.86 (*SEM = *.02) for the control group and.66 (*SEM = *.04) for the experimental group. The participants in the control group generated a higher P(Cause) than the participants in the experimental group. There was also an increase in the P(Cause) for the positive contingency condition relative to the zero contingency condition. A 2 (experimental vs. control groups) × 2 (zero contingency vs. positive contingency) ANOVA for the P(Cause) showed the significant main effects of Group, *F*(1, 58) = 17.88, *MSE* = .07, *p*<.001, η_p_^2^ = .24, and Contingency, *F*(1, 58) = 15.08, *MSE = *0.02, *p*<.001, η_p_^2^ = .21. The interaction was not significant, *F* <1. As expected, the participants that were exposed to the intervention generated a lower P(Cause) than participants in the control group. There was also an unexpected main effect of Contingency, showing that the participants introduced the target cause in a higher proportion of trials in the positive contingency condition than in the zero contingency condition. We could hypothesize that this effect might be related to the fact that administering the medicine produced a higher relief rate than not administering the medicine in the positive contingency condition. Even though the participants were instructed to try to determine whether the medicine was effective (instead of producing the relief in as many patients as possible), given that relief can be considered a desirable outcome, they may have found it difficult to refrain from acting in a way that increased their chances of curing the fictitious patients.

**Figure 2:**
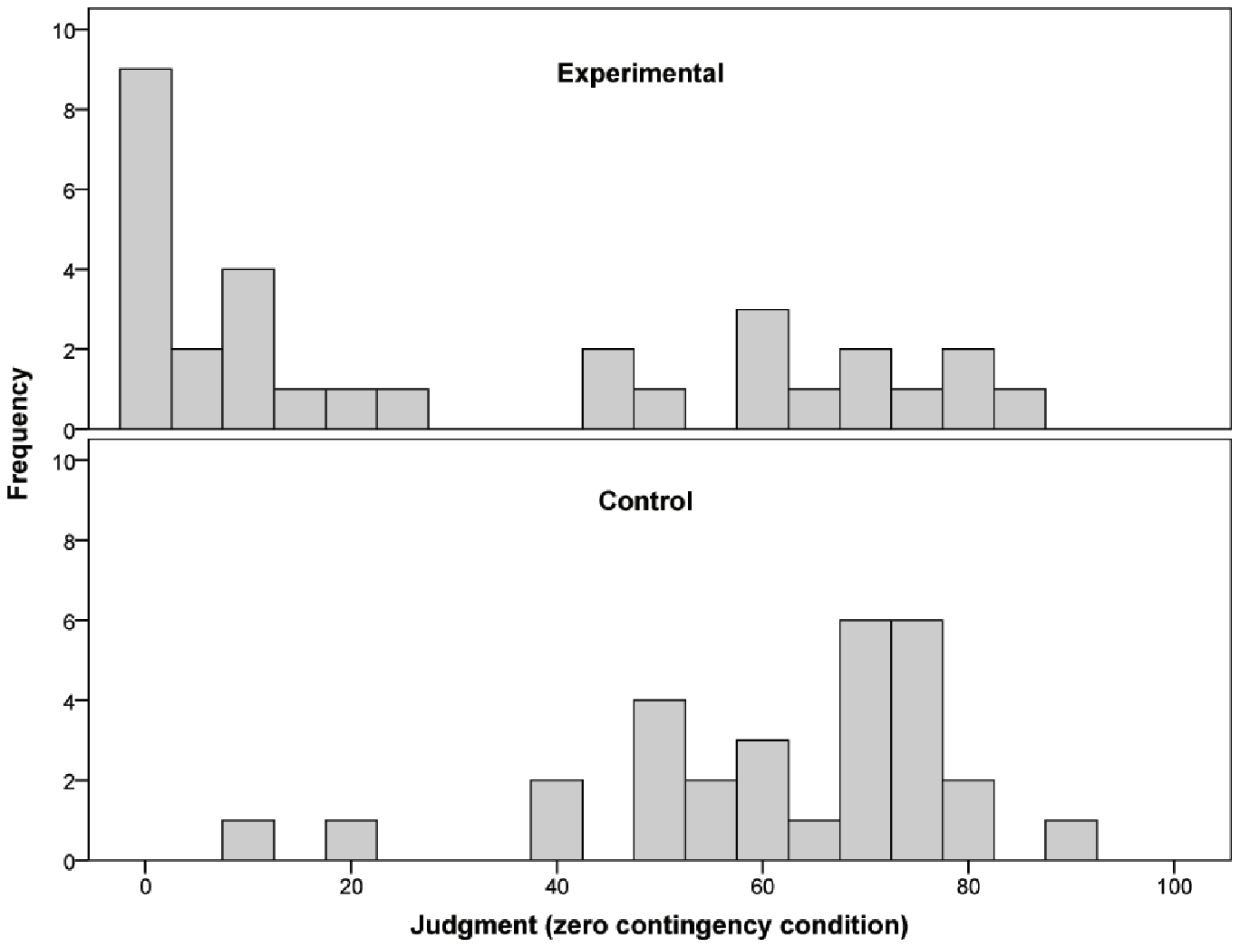
Histogram depicting the distribution of the judgments in the zero contingency condition, for the experimental group (top panel) and the control group (bottom panel).

The bottom panel of Figure 1 shows the causal judgments made by the participants at the end of the task. The values were high and similar for the two groups in the positive contingency condition: the mean judgment was 70.28 (*SEM* = 2.77) for the control group and 71.81 (*SEM* = 2.59) for the experimental group. By contrast, in the zero contingency condition, the judgments made by the participants in the experimental group were closer to zero (i.e., they were more accurate) than those made by the participants in the control group: the mean judgment was 61.69 (*SEM* = 3.35) for the control group and 30.55 (*SEM* = 5.59) for the experimental group. Therefore, the intervention reduced the illusory perception of causality, leading the participants in the experimental group to make more realistic judgments than the control participants. This conclusion is supported by the information conveyed by the histograms in Figure 2: the intervention led many participants in the experimental group to give a judgment of zero in the zero contingency condition. A 2 (experimental vs. control groups) × 2 (zero contingency condition vs. the positive contingency condition) ANOVA on the judgments of causality showed significant main effects of Group, *F*(1, 58) = 16.34, *MSE = *401.98, *p*<.001, η_p_^2^ = .22, and Contingency, *F*(1, 58) = 39.55, *MSE = *470.58, *p*<.001, η_p_^2^ = .41. More importantly, there was a significant interaction between Group and Contingency, *F*(1, 58) = 16.99, *MSE = *470.58, *p*<.001, η_p_^2^ = .23. The causal judgments did not differ significantly between the experimental group and the control group for the positive contingency, *t*(58) = 0.40, *p = *.69, but they did differ for the zero contingency, *t*(58) = 4.70, *p*<.001. Thus, the participants in the experimental group were able to detect the existence of a causal relationship when there was good evidence for it and to detect its absence when there was no evidence for it.

In addition, analyses were conducted to determine whether the effect of the intervention on the judgments in the zero contingency condition was direct or whether it was mediated by the differences in P(cause) between the two groups. Thus, we conducted a mediation analysis [56] to isolate the direct effect of Group (i.e., the intervention) on judgments while partialling out the effect of the P(Cause). In other words, we can determine the amount of variance in causal judgments between the experimental and control groups that can be attributed to the intervention producing differences in the P(Cause) between the two groups, which, in turn, produced the differential perceptions of causality (see [34] for a similar strategy).

**Figure 3:**
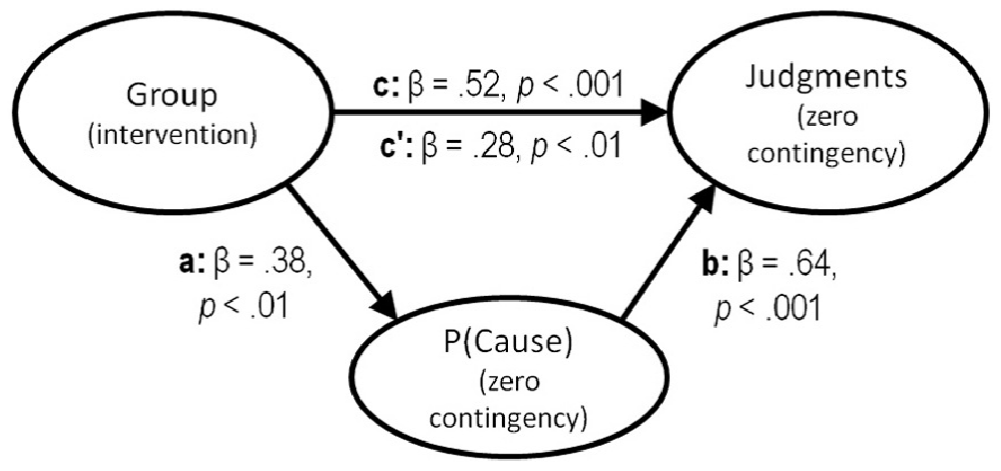
Mediational structure underlying the experimental manipulation in the zero contingency condition. The total effect of the intervention on the causal judgments, depicted as path c, is divided into two components: one indirect effect (paths a and b) through the P(Cause), and one direct effect (path c'), which is the result of discounting the indirect effect. The mediation analysis reveals that the intervention affected the judgments both directly and indirectly, via the P(Cause).

The mediation analysis procedure described by Baron and Kenny [56] consists of three consecutive steps that reveal three pieces of information: the total effect of Group on judgments (path c in Figure 3), the indirect effect explained by the mediation of P(Cause) (paths a and b in Figure 3 ), and the direct effect of Group that remains after the indirect effect has been partialled out (path c' in Figure 3). Following this procedure, we first assessed the total effect of Group on the judgments (by regressing the judgments onto the Group), β = .52, *t*(58) = 4.70, *p*<.001. Next, we assessed the indirect effect of Group on judgments mediated by the P(Cause). This involves two requirements: (a) ensuring that the participants in the experimental group, compared with those in the control group, did generate a lower P(Cause) [i.e., the P(Cause) was regressed onto the Group], β = .38, *t*(58) = 3.09, *p*<.01; and (b) showing that the P(Cause) had a positive impact on judgments while controlling for the effect of Group [this was done by conducting a multiple regression analysis on the judgments with P(Cause) and Group as predictors], β = .64, *t*(57) = 7.32, *p*<.001. Ultimately, the same multiple regression model [with P(Cause) and Group as predictors of judgments] revealed that a significant direct effect of Group on judgments remained after the indirect effect mediated by the P(Cause) had been partialled out, β = .28, *t*(57) = 3.26, *p*<.01. The Sobel test [57] indicated that, although it was significant, the direct effect of Group on judgments was significantly smaller than the total effect reported in the first step in which the effect of P(Cause) was not partialled out, *z* = −2.85, *p*<.01. This partial mediation suggests that the impact of the intervention on causal judgments in a zero contingency situation occurred for two reasons: first, the intervention directly produced more realistic judgments in the experimental group; second, the intervention also affected the judgments indirectly by decreasing the P(Cause) that the participants generated.

It could be argued that, since the participants decided the number of trials in which the potential medicine was administered, the actual contingency experienced by participants could slightly depart from the programmed contingency. However, these variations were minimal: the mean contingency to which the participants were exposed in the zero contingency condition was.04 (*SEM* = .04) for the control group and.02 (*SEM* = .02) for the experimental group, whereas the mean contingency to which the participants were exposed in the positive contingency condition was.63 (*SEM* = .04) for the control group and.64 (*SEM* = .01) for the experimental group. Nevertheless, and in order to ensure that these variations did not affect the results, we repeated all the previous statistical analyses on accuracy scores instead of causal judgments, finding the same conclusions. Accuracy was computed as the absolute difference between the causal judgment (rescaled to range between 0 and 1) and the actual contingency each participant was exposed to.

## Discussion

In the Introduction, we suggested that causal illusions underlie many of the superstitious and pseudoscientific beliefs that prevail in our society [7]. Because unrealistic beliefs can be extremely harmful, we believe that teaching people how to evaluate causal hypotheses more accurately has broad implications for the effort to develop a knowledge-based society. The present intervention constitutes an initial effort in this direction.

We found that training a group of adolescents in the rational manner of making inferences about cause-outcome relationships decreased their illusory perceptions of causality in a subsequent non-contingent situation. Moreover, including a control condition in the positive contingency scenario allowed us to conclude that the lower causal ratings observed in the experimental group could not be solely explained by a general increase in suspicion in this group. Rather, the group specifically made more realistic judgments in the null contingency condition while preserving an accurate view of the positive contingency condition.

In addition, a mediation analysis showed that one of the reasons for this decrease in the illusion of causality was that the intervention helped the participants to diminish their exposure to the potential cause. As noted in the Introduction, our spontaneous tendency is to expose ourselves to more cause-present observations than cause-absent observations, which strongly contributes to the development of the illusion [24]. As shown in Figure 1, the behavior of the participants in the control group supported the idea that this spontaneous tendency is the default strategy: the participants in this group generated an average P(Cause) of.75 (i.e., they chose to administer the medicine in approximately 3 of 4 observations) when the situation involved a zero cause-outcome contingency. In contrast, the behavior of the participants in the experimental group suggests that they internalized the importance of experimental control because they tended to generate more cause-absent observations than did those in the control group. The average P(Cause) in the experimental group in the zero contingency condition was only .56.

In addition to the differences between the information sampling strategies, the causal judgments reported by the participants in the experimental group were lower than those reported by the control group. Although the mediation analysis reported above showed that the effect of the intervention on causal judgments was partially mediated by differential exposure to cause-present trials, the intervention still had a significant, direct effect on the final causal judgments that was independent of the P(Cause) that the participants generated. This effect is consistent with the idea that people tend to spontaneously weight cause-present information (and especially cause-outcome coincidences) more heavily than they do cause-absent information [30]. On this basis, it seems that the intervention most likely affected causal judgments in two different ways: first, by diminishing the P(Cause) generated by the participants, and second, by encouraging the participants to pay more attention to cause-absent information, P(Outcome|∼Cause), or to weight that information more heavily. Future studies should measure the effectiveness of the debiasing intervention while controlling the subsequent exposure to the P(Cause). This could be easily done by employing observational contingency learning tasks (e.g., [7]) in which participants cannot decide if the cause is introduced or not but they merely observe whether the cause is present and whether the outcome occurs [i.e., the P(Cause) is set up by the experimenters]. This would allow us to isolate the individual influence of the intervention in the evidence sampling strategies from its influence in the interpretation of this evidence.

Our approach to measuring the effectiveness of the intervention was fairly different from the conventional strategy that is used in the statistical reasoning literature [55], [58], [59]. These studies typically measure the internalization of statistical concepts by presenting the participants with verbal descriptions of everyday situations that involve applying these concepts. For example, Fong et al. [55] trained a group of participants in the “law of large numbers” and found improvement when the participants were asked to reason about verbal descriptions of new examples to which the law was relevant. The analogous strategy in our domain could be presenting participants with information about the relevant probabilities for calculating the contingency, P(Outcome|Cause) and P[Outcome|∼Cause), in new scenarios and then determining whether they could infer that there was (or that there was no) good empirical evidence for assuming a causal relationship. Instead, in the present work, the effectiveness of the intervention was measured using a transfer task that required the practical application of the principles underlying the concept of contingency in a trial-by-trial setting. Our study suggested that understanding the necessity of considering the P(Outcome|∼Cause) value led to a change in the decision-making process on a trial-by-trial basis. We believe that this strategy is meaningful because the contingency learning task entails a context that resembles more closely the process of causal induction in many real life situations in which people (and other animals) learn by sequential observations of the presence and absence of the potential cause and the outcome over time, instead of encountering the covariational information in a summarized format. The use of a sequential presentation format also has theoretical implications; it has been suggested that the cognitive mechanisms that operate when dealing with trial-by-trial information (such as the information in our study) are not necessarily the same as those that are activated when this information is received in a summarized form [60]. Finally, an additional advantage of using a contingency learning task to assess the intervention is that this is a standard procedure in the study of human judgment and decision-making, and thus, an extensive body of relevant theoretical and experimental literature is available [61]. Nevertheless, in our modern society people frequently have to deal with summarized information (e.g., statistics that appear in the newspapers) and, therefore, future studies might explore if the present results prevail when employing a summarized version of the contingency learning task, in which participants would receive verbal descriptions of the probabilities involved in the situation.

We noted in the Introduction that the spontaneous tendency to generate a high P(Cause) could be related to a more general information sampling bias that some authors have called a positive testing strategy [35]. For a hypothesis involving a potential cause-outcome relationship between two events, this strategy may involve preferentially testing the hypothesis by searching for cases in which the outcome would occur if the hypothesis were correct (i.e., situations in which the potential cause is present). A positive test strategy can be categorized as a form of “strategy-based error” [47], [48], a type of error that is relatively conscious or deliberative [48], [62]. Because of its deliberative nature, people who fall prey to this type of error might be especially good candidates for debiasing when the benefits of accuracy are stressed or when the stakes for correctness are high enough [47]. Thus, the success of our debiasing intervention could at least be partially attributable to our emphasis on the benefits of accurate causal estimation. To emphasize these benefits, we employed an initial phase in which the participants were deceived in a manner that could also occur in their daily lives (i.e., the presentation and demonstration of a “miracle product”). This first phase was also intended to help us avoid a potential problem acknowledged by some authors, such as Lilienfeld et al. [10], who have stated that participants might not benefit from debiasing efforts because they have trouble perceiving their own biases [50] and therefore fail to realize that they need a remedy. Thus, the initial staging phase was also aimed to increase the participants’ awareness of how easily causal illusions can develop. Although we did not formally assess the success of this specific aspect of the intervention, when the participants were asked at the end of the session, the majority of them expressed that they were cheated in the first phase. However, at present, we cannot elucidate whether the intervention would have been equally effective if we had simply described the abstract concept of contingency or, more generally, whether omitting some components of the intervention would have affected the results. Future studies should also explore which features of the intervention are crucial or contribute to its effectiveness to a greater extent.

To this respect, the reason for using a between-subjects instead of a within-subjects design (the latter would involve measuring the same participants’ performance before and after the intervention) was to unambiguously determine the influence of the intervention apart from the potential effect of the practice or familiarity with the same contingency learning task (involving the same contingencies). We consider that this is an appropriate starting point for the first attempt to debias adolescents against causal illusions. Nevertheless, future interventions should also include within-subjects measures of the effectiveness of the intervention (i.e., pretest-posttest designs), together with active control groups that would require subjects to engage in alternative causal inference tasks different from the intervention presented here.

As far as we know, the present study is the first serious effort to implement and experimentally evaluate an educational intervention to prevent the formation of causal illusions. We believe that the potential benefits of this type of intervention are considerable. Causal illusions may underlie many harmful beliefs that eventually generate important societal issues (e.g., the use of pseudo-medicines, racism, economic collapse) and too often guide political decisions. Unsurprisingly, many governmental and scientific organizations are now committed to advancing scientific reasoning and literacy at school [63], [64]. Because our current study was conducted in a classroom in a school context, we feel confident that this type of evidence-based intervention could be easily implemented in an educational program. In addition, the fact that our evaluation was based on a standardized method used in experimental psychology makes it an ideal tool for assessing and comparing different debiasing strategies in different schools. Indeed, developing a standardized method that can be used for comparison purposes will be the first step toward a truly evidence-based debiasing program worldwide.

As stated by Lilienfeld [65], “…the most important psychological experiment never done would […] begin with the construction of a comprehensive evidence-based educational programme of debiasing children and adolescents in multiple countries against malignant biases…”, and “…launching such an endeavour by conducting small-scale pilot studies would seem to be a worthwhile starting point". To the best of our knowledge, the “most important experiment” for “sav[ing] the world” (as Lilienfeld phrases it) remains to be conducted, but we believe that the intervention and the assessment procedure presented here constitute a promising advance in this direction.
